# Recent Advances in the Prediction of Fouling in Membrane Bioreactors

**DOI:** 10.3390/membranes11060381

**Published:** 2021-05-24

**Authors:** Yaoke Shi, Zhiwen Wang, Xianjun Du, Bin Gong, Veeriah Jegatheesan, Izaz Ul Haq

**Affiliations:** 1 Department of Automation, College of Electrical and Information Engineering, Lanzhou University of Technology, Lanzhou 730050, China; yaoke_shi@163.com (Y.S.); wwwangzhiwen@163.com (Z.W.); gong_bin01@163.com (B.G.); Izaz.lut@gmail.com (I.U.H.); 2 Key Laboratory of Gansu Advanced Control for Industrial Processes, Lanzhou University of Technology, Lanzhou 730050, China; 3 National Demonstration Center for Experimental Electrical and Control Engineering Education, Lanzhou University of Technology, Lanzhou 730050, China; 4 School of Engineering, RMIT University, Melbourne 3000, Australia; jega.jegatheesan@rmit.edu.au

**Keywords:** artificial neural network, mathematical model, membrane bioreactor, fouling prediction

## Abstract

Compared to the traditional activated sludge process, the membrane bioreactor (MBR) has several advantages such as the production of high-quality effluent, generation of low excess sludge, smaller footprint requirements, and ease of automatic control of processes. The MBR has a broader prospect of its applications in wastewater treatment and reuse. However, membrane fouling is the biggest obstacle for its wider application. This paper reviews the techniques available to predict fouling in MBR, discusses the problems associated with predicting fouling status using artificial neural networks and mathematical models, summarizes the current state of fouling prediction techniques, and looks into the trends in their development.

## 1. Introduction

**Figure 1:**
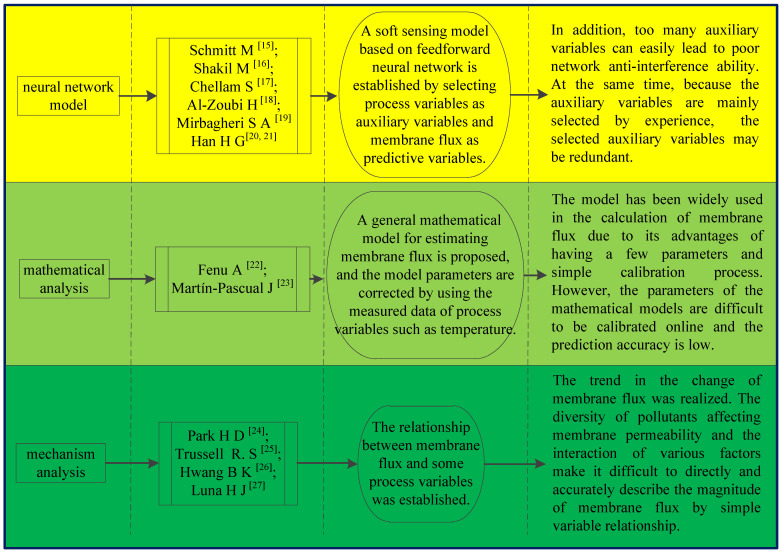
An overview of fouling prediction methods [15,16,17,18,19,20,21,22,23,24,25,26,27].

Under the circumstances where water pollution is a great concern and the increasing demand for treatment efficiency and effluent quality are of paramount importance, membrane bioreactors (MBR) [1] have been widely used, based on their merits in addressing those issues. MBR technology is recognized globally as one of the most potential high-tech applications in the field of water treatment in the 21st century. MBR is a wastewater treatment system that combines membrane technology and biological treatment technology [2], and mainly composed of membrane modules and bioreactors [3,4,5]. It does not need secondary clarification and has the advantages of small area requirement, good effluent quality, and low sludge production. However, it also has the disadvantages of high cost, high energy consumption, and its membranes can be fouled easily [6,7]. Membrane fouling can shorten membrane life and cause unnecessary loss of productivity, which is one of the important reasons limiting the development of MBR for wider applications [8]. Before membrane fouling occurs, timely cleaning or replacement of membrane modules can effectively prolong the service life of the membranes and reduce operating costs [9,10]. Therefore, it is very important to predict membrane fouling and in order to do so factors such as membrane flux, transmembrane pressure (TMP), related operating conditions and predicting their correlation need to be researched [11,12,13,14]. The authors reviewed the literature on MBR membrane fouling prediction published worldwide and found that there is scarce literature on the prediction of fouling of membranes in MBRs. Therefore, research outcomes on the prediction of membrane fouling are reviewed in this work, which hopes to be helpful for researchers conducting further study in the future. Figure 1 is an overview of fouling prediction methods.

## 2. Method Based on Artificial Neural Networks to Predict Membrane Fouling

**Figure 2:**
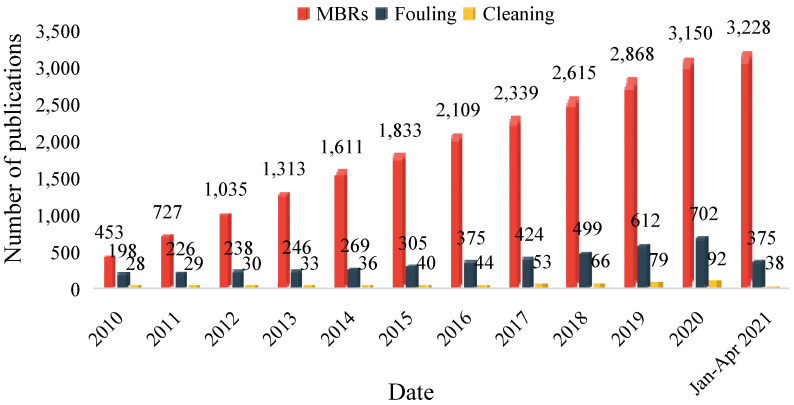
Number of papers published between 2010 and 2021 on MBRs and their fouling and cleaning.

As a mathematical processing method to simulate the structure and function of the biological neural system, an artificial neural network (ANN) has the ability to learn from existing information on the inputs of a process and produce results similar to the ones obtained from the process. It includes three main parts: an input layer of neurons, a hidden layer of neurons, and an output layer of neurons, and ANN is often used to solve problems such as classification and regression [28]. After years of research and exploration, it has been found to have a strong advantage in the field of prediction. The prediction method based on ANN aims to take the original measurement data or the features extracted from the original measurement data as the input of the network, to continuously adjust the structure and parameters of the network through certain training algorithms, and to use the optimized network for prediction. No prior information is needed in the prediction process, and the prediction results are completely based on the monitoring data [29]. For the complexity of membrane fouling prediction, ANN is undoubtedly a simple method, which can connect the input variables in wastewater treatment operation to the membrane fouling status without any mechanistic equations [30]. The simple structure of ANN makes its application and processing simple and efficient. However, when the input data is not prepared well enough or the structure is too complex, the neural network used to predict membrane fouling is easy to be overfitted. In fact, collecting a large number of data increases the processing time and the operating costs [31]. Meanwhile, an over-optimized ANN will also easily lead to overfitting [32]. Therefore, scholars worldwide have carried out much research and tried to find a balance between performance and feasibility. Some studies have focused on the development of ANNs considering pilot-scale MBRs, and achieved good results in predicting the TMP, permeability, and flux of a MBR. To apply ANN to the prediction of membrane fouling in a large-scale membrane bioreactor, it is necessary to carry out in-depth research on this subject. The number of papers published in this area between 2010 and 2021 using Baidu Scholar search engine is shown in Figure 2. The average annual growth rate since 2010 is 22.7%.

As early as 2000, some scholars began research on ANN-based membrane fouling prediction. Dornier et al. [33] first applied ANN to membrane fouling and successfully predicted the fouling of membrane by sugarcane juice diluent filtered by a ceramic microfiltration membrane under short-term operations. However, the selection of data for network model learning and validation needed further research. Then, back-propagation neural network (BPNN) predicted the membrane fouling of municipal drinking water filtered by nanofiltration membrane, and the network took the total resistance as the prediction output to characterize the status of membrane fouling [34]. The TMP difference can reveal membrane fouling to a great extent while the membrane filtration resistance characterizes it. ANN is used to study the variation trend in the TMP difference before and after backwashing, and a recurrent neural network model for the long-term behavior of membrane filtration was established. After inputting the operating conditions, as long as an initial resistance of membrane filtration is an input, the membrane filtration resistance value in the following days can be predicted accurately, which can be used to predict the status of membrane fouling [35].

Since then, the prediction of membrane fouling in different types of membrane bioreactors has been verified with the development of ANNs. Schmitt et al. [15] used an ANN model to predict TMP difference in the process of an anoxic–aerobic membrane bioreactor (AO–MBR) treating domestic wastewater. Ten parameters related to wastewater treatment measured at different locations of the AO–MBR system were used as input variables of the ANN model, which verified that ANN was an effective way to predict membrane fouling of an AO–MBR system treating domestic wastewater.

Another study used ANNs to predict the flux evolution of MBR on a pilot scale [36]. The wastewater treatment system consisted of two different MBR units. Each unit had three hollow fiber membrane modules. An Elman network was used for modeling, which is composed of a standard feed-forward neural network (FFNN) with a single hidden layer, only adding a feedback connection between the output of the hidden layer and its input. The permeate flux was the output of the ANN model, and the input data include TMP, rate of change of TMP, TMP during the backwash, length of filtration cycle and backwash, sludge retention time (SRT), total suspended solids (TSS), temperature, and oxygen decay rate in the aerobic zone. During the experiment, TMP increased gradually, ranging from 0 to 40 kPa. The average deviation was only 2.7% after modeling the permeate flux. However, the parameters related to wastewater characteristics or mixture were not considered in their work, which leads to a lack of reliability in predicting membrane fouling. Moreover, due to the high number of hidden neurons, the predicted value of permeate flux may exceed the experimental data. The problem of over fitting will appear when there are more hidden layers. At the same time, it will also make the training and the convergence of the model difficult.

In recent years, soft sensing technology has been used widely in the identification and prediction of variables in the wastewater treatment process based on the characters of economic reliability, rapid dynamic response, and high accuracy of identification and prediction. This has become a new approach in membrane flux prediction [16,37]. Chellam [17] and Al-Zoubi et al. [18] obtained multiple process variables related to membrane permeability by analyzing the mechanisms, and 29 of them were selected as auxiliary variables to establish a soft sensing model of membrane permeability based on BPNN. The prediction accuracy reached 70%. However, due to the selection of too many auxiliary variables and the large initial scale of the network, the learning time of the network was very long. Too many auxiliary variables also led to the poor anti-interference ability of the network. Therefore, it can only be used in the pilot platform, and cannot be applied in the practical application of wastewater treatment plants. Mirbagheri et al. [19] selected six of the process variables, including chemical oxygen demand (COD), concentration of sludge, and SRT, as the auxiliary variables, and the membrane flux as the output variable to establish the radial basis function (RBF) model. The RBF-based soft sensing model can successfully predict the permeability of the membrane. However, because the auxiliary variables are mainly selected by experience, the selected auxiliary variables may be redundant, and the prediction accuracy of the model was still unable to meet the actual requirements.

In the aspect of optimizing the structure of ANN, an optimized BPNN-based prediction model was established to predict the ceramic membrane fouling of aqueous extract of Chinese medicine, in which the optimal number of neurons in the hidden layer, the connecting weights and the thresholds of the network model were improved by using an optimization algorithm [38]. In the experimental process, 207 groups of Chinese medicine aqueous extract data were extracted for network training and prediction. Comparatively, the performance of the improved BPNN model is more stable than the traditional BPNN, the RBF network and the multiple regression analysis. The success rate in reaching the preset goal in 20 random running experiments was as high as 95%, which can be adapted to the multi-dimensional and nonlinear data collected in the process of ceramic membrane purification of Chinese medicine aqueous extract. It can be used to predict the degree of membrane fouling stably and accurately, which provides an effective method for the prediction and prevention of fouling of ceramic membrane used to filter the aqueous extracts of Chinese medicine. However, the effectiveness of this prediction model for different raw water has not been mentioned, and needs to be further verified.

To effectively and accurately predict membrane flux of MBR, an improved extreme learning machine (ELM) prediction model was proposed by Yang and Li [39]. Aiming at overcoming the shortcomings of BPNN, such as easily falling into local minimum and poor generalization performance, ELM can effectively overcome these and can obtain good generalization performance at extremely high speed. Since the input weights and hidden layer thresholds are given randomly, ELM usually needs more hidden layer nodes to achieve the desired accuracy [40]. Particle swarm optimization (PSO) was used to optimize the weights and thresholds of ELM to establish the prediction model of PSO-ELM. The principal components extracted by a principal component analysis (PCA) dimension reduction algorithm was used as the input of the model, and the membrane flux was used as the output of the model. The results show that the model has better generalization ability and higher prediction accuracy for MBR membrane flux prediction. In their study, three variables are selected as inputs, including the influent pressure, effluent pressure and corrected flow rate, while other important factors such as mixed liquid suspended solids (MLSS) and temperature were not considered. Therefore, the practicability of the model was studied by Tang et al. [41]. They first used the PCA method to determine the main factors which had significant influence on membrane fouling, including MLSS, operating pressure, and temperature. Then, a prediction model based on the RBF neural network was established. To some extent, high prediction accuracy depends on the parameter selection of network model [42]. Hence, a genetic algorithm (GA) was introduced to the model to optimize the parameters and achieve good global performance. Finally, the prediction results were compared with the measured data. The results illustrated that the convergence speed and prediction accuracy of the membrane fouling simulator based on GA-RBF was better than the RBF network, and the expected goal was achieved. The whole experimental process had a certain theoretical value and practical significance, which would play a positive role in guiding the practical application of MBR.

Li et al. introduced an improved PSO algorithm to the fuzzy RBF neural network to give it a strong nonlinear approximation ability, a self-learning ability, an adaptive ability, and a transient/steady-state performance [43]. Tao et al. employed an improved PSO algorithm to train the fuzzy RBF neural network [44]. The initial weights and thresholds of the fuzzy RBF neural network are obtained by using the improved PSO algorithm, and then the final weights and thresholds are obtained by a secondary optimization. Their results showed that the PSO-based fuzzy RBF neural network is feasible for MBR membrane fouling prediction, which shortens the response time, has a small steady-state error, and can better fit with the actual membrane flux and better predict this.

Based on the RBF neural network, Han et al. [20] proposed a soft sensing method based on a recurrent radial basis function neural network (RRBFNN). Firstly, based on the actual operating data of a wastewater treatment process, the partial least squares (PLS) method was applied to screen out the process variables related to membrane permeability. Secondly, the soft sensing model of membrane permeability was established based on RRBFNN, and the parameters of RRBFNN were adjusted by fast gradient descent algorithm to ensure the accuracy of the soft sensing model. Finally, the designed soft sensing model of membrane permeability was applied to the actual sewage treatment process, and the model was verified by the measured data of wastewater treatment plant. Results showed that the soft sensing model can accurately predict membrane permeability and has a good prediction accuracy. The parameters to be adjusted are output weight w(t), feedback weight u(t), the center of hidden layer neuron c(t) and the width of hidden layer neuron σ(t). The updated formula is:

(1) $$\{\begin{array}{c} c\left(t+1\right)=c\left(t\right)-\eta_{1}\varphi_{c}\left(t\right)w\left(t\right)e\left(t\right)\\ \sigma \left(t+1\right)=\sigma \left(t\right)-\eta_{2}\varphi_{\sigma }\left(t\right)w\left(t\right)e\left(t\right)\\ u\left(t+1\right)=u\left(t\right)-\eta_{3}\varphi_{u}\left(t\right)w\left(t\right)e\left(t\right)\\ w\left(t+1\right)=w\left(t\right)-\eta_{4}\varphi_{w}\left(t\right)e\left(t\right) \end{array}$$

where $\eta_{1}$, $\eta_{2}$, $\eta_{3}$, $\eta_{4}$ are the learning rates of c(t), σ(t), u(t) and w(t) respectively; and e(t) is the difference between the actual output and the calculated output of the neural network at time t. When the neural network is initialized, the network parameters are set to random values. After the network initialization, the parameters of the neural network are judged and adjusted according to the error value. The calculation formulae of $\varphi_{c}\left(t\right)$, $\varphi_{\sigma }\left(t\right)$, $\varphi_{u}\left(t\right)$, and $\varphi_{w}\left(t\right)$ are:

(2) $$\{\begin{array}{c} \varphi_{c}\left(t\right)=\partial \theta \left(t\right)/\partial c\left(t\right)\\ \varphi_{\sigma }\left(t\right)=\partial \theta \left(t\right)/\partial \sigma \left(t\right)\\ \varphi_{u}\left(t\right)=\partial \theta \left(t\right)/\partial u\left(t\right)\\ \varphi_{w}\left(t\right)=\theta \left(t\right)-\varphi_{c}\left(t\right)c\left(t\right)-\varphi_{\sigma }\left(t\right)\sigma \left(t\right)-\varphi_{u}\left(t\right)u\left(t\right) \end{array}$$

The advantage of the fast gradient descent algorithm is that when there are many data to be processed, the calculation speed of neural network will not reduce, and this can ensure the convergence of the algorithm, which is suitable for the use of practical sewage treatment problems. The network output error is:

(3) $$\left(t\right)=y\left(t\right)-y_{d}\left(t\right)$$

where $y_{d}\left(t\right)$ is the measured data at time t.

The mechanistic mathematical model [45,46,47,48] and computational fluid dynamics simulation [49,50] show excellent performance in a large number of simulation studies of transmembrane pressure difference and flux. However, the complexity of membrane fouling limits the development of these models. To obtain practical kinetic equations, it is necessary to simplify the operating conditions and wastewater characteristics. On this basis, an ANN model based on a recurrent network is proposed to predict the evolution of hydraulic resistance, sediment thickness, and infiltration flux respectively, with TMP and other inflow characteristics as inputs [51,52]. However, in these neural network models, only one set of initial input values is used to predict the whole output set. Geissler et al. [53] used an ANN model to predict the changing trend of membrane flux in MBR. The input parameters are TMP, rate of change of TMP, length of filtration cycle and backwash cycle length, SRT, total suspended solids, aerobic zone temperature, and oxygen decay rate, while the output is membrane flux. Other important parameters such as COD, dissolved oxygen (DO) and MLSS were not considered in their research. In fact, due to a large number of hidden neurons, the predicted value of membrane flux may be over-fitted.

A literature review found that research on predicting membrane fouling in forward osmosis membrane filtration process by the ANN is rarely exercised. Jawada et al. [54] constructed a multilayer neural network model to predict the permeation flux of forward osmosis. The model studied the influence of the number of neurons and hidden layers on the performance of the neural network, which is helpful in optimizing the development of the network structure. The coefficient of determination (*R*^2^) value of the optimized network is as high as 97.3%, which shows the effectiveness of the model in predicting the target yield. Moreover, the effectiveness and generalized prediction ability of the model are verified by some untrained data. The performance of the neural network model is compared with the mass transfer model and multiple linear regression (MLR) model. The results show that the proposed model is superior in the prediction of fouling in the forward osmosis membrane.

The ANN based prediction model can achieve better accuracy by avoiding problems such as the determination of network topology and local extremum value [55]. Based on statistical learning theory and structural risk principle, the support vector machine (SVM) maps the problem to be solved into a high-dimensional space and transforms it into a quadratic optimization problem, which solves the local extremum problem of the neural network. Compared with the traditional SVM, the least squares support vector machine (LSSVM) transforms inequality constraints into equality constraints and transforms the solving process into solving a group of equations, which significantly speeds up, relatively [56]. However, the problem of parameter selection of LSSVM has seriously hindered its development. Nie et al. [57] used the GA algorithm for parameter selection of LSSVM and proposed an MBR membrane flux prediction algorithm based on a GA based LSSVM. To select the parameters of LSSVM accurately, GA is used to optimize the penalty factor and kernel function parameters of the LSSVM model. Meanwhile, PCA was used to determine the main factors that affect MBR membrane flux. The important factors were extracted as the input layer of LSSVM, and the membrane flux was taken as the output. The GA-LSSVM prediction model had a high prediction accuracy for membrane flux.

Zhou et al. [58] established a BPNN model for single-step prediction based on data interpolation and multi-step memory, with the influent water quality concentration of BOD, total nitrogen (TN), total phosphorus (TP), sludge concentration, and influent flow as the auxiliary variables. This model was used to predict the BOD, TN, and TP in the effluent. However, it was only capable of single-step prediction of the parameters mentioned above.

During wastewater treatment, the membrane flux is often used to evaluate the fouling of membrane, and a multi-step prediction of the permeability rate can bring more economic benefits. Therefore, Han et al. [21] proposed a multi-step prediction method of MBR permeability rate based on FNN. Moreover, a soft sensor model was established using the improved Levenberg Marquardt (L-M) algorithm to adjust and optimize the center, width, output weight, and other parameters in a fuzzy neural network (FNN) [59]. In this study, auxiliary variables are taken as the inputs of FNN, while permeability was considered as the output. A multi-step prediction method based on time difference (TD) is proposed to achieve high accuracy. With this method, error accumulation when predicting auxiliary variables is alleviated, and the prediction accuracy is improved. Multi-step prediction is a strategy to achieve value predictions by iterating the single-step prediction procedure, which aims to obtain the output at time step (t+1) time from the variables at time step t:

(4) $${\hat{x}}_{t+1}=f\left(x_{t}\left(1\right),\;x_{t}\left(2\right),\ldots ,x_{t}\left(n\right)\right)$$

where, ${\hat{x}}_{t+1}$ is the predicted output at time t+1, and $x_{i}\left(1\right),\;x_{i}\left(2\right),\ldots ,{\hat{x}}_{t+1}$ are n observations at time t.

Indirect multi-step prediction integrates the output of single-step prediction into the input for the subsequent calculation:

(5) $${\hat{x}}_{t+m}=f\left(x_{t}\left(1\right),\;x_{t}\left(2\right),\ldots ,x_{t}\left(n\right),{\hat{x}}_{t+1},\ldots ,{\hat{x}}_{t+m-1}\right)$$

The TD method combines dynamic programming and Monte Carlo sampling algorithms. It is capable of updating the function values in a single and rapid step. The updating formula of the TD method is as follows.

(6) Z(t+1)=Z(t)+α(G(t)−Z(t))

where Z is the auxiliary variable, α is the deviation, and G(t) is the updated return value.

Altunkaynak et al. [60] established a soft sensing model of water permeability based on a fuzzy neural network (FNN). In the model, initial flux, inlet shear rate, instantaneous pressure, and filtration time were selected as auxiliary variables based on the mechanism analysis. The model was based on fuzzy mathematics and produces uncertain results.

The osmotic membrane bioreactor (OMBR) is a new technique for wastewater treatment, but its greatest challenge is membrane fouling. The membrane flux of OMBR was simulated and predicted by the adaptive network-based fuzzy inference system (ANFIS) and ANN models based on the adaptive network [61]. MLSS, EC, and DO were used as the model inputs. The ANFIS model and the ANN models were used to predict the parameters of thin-film composite (TFC) and cellulose triacetate (CTA) membranes, and the models’ R^2^ were 0.9755 and 0.9404 for TFC and 0.9861 and 0.9817 for CTA, respectively. The root means square errors of TFC (0.2527) and CTA (0.1230) in the ANFIS model were lower than those in the ANN model, which were 0.4049 and 0.1449, respectively. Besides, RMSE, SSE, Adj-R^2^, and R^2^ were used to compare the two established models, and the results showed that the ANFIS model possessed better prediction ability than the ANN model [62].

To solve the problem of membrane fouling, a membrane cleaning decision model was established with membrane recovery as the decision indices. In this model, Bandelet transform was combined with a neural network, creating a Bandelet neural network to predict membrane flux and recovery. However, the Bandelet neural network still has some limitations, such as its high sensitivity to initial weights, its tendency to fall into the local optimal solution during optimization, and the overfitting problem [63,64].

Zhao et al. [65] combined Bandelet transform with a neural network and designed a Bandelet neural network to predict membrane flux and recovery rate so that reasonable decisions on membrane cleaning could be made. Specifically, Bandelet function and scale function were used as the activation functions of the hidden layer and the output layer, respectively. Besides, the improved Bat algorithm [66] was integrated in the Bandelet neural network to improve the optimization outcome. The model is advantageous in its prediction accuracy and speed, and the prediction results are better than those produced by other prediction models.

## 3. Prediction of Membrane Fouling Based on Mathematical Models

In the current prediction models of membrane fouling during coagulation–membrane filtration, the extended Derjaguin-Landau-Verwey-Overbeek (XDLVO) theory has generally been used to calculate the activation energy of smooth interfaces, but the surface morphology of coagulation flocs will have a greater impact on the prediction results than the activation energy [67]. The sine wave sphere model was used to simulate the surface of rough humic acid (HA) flocs, and a strategy combining surface element integration (SEI), XDLVO theory and composite Simpson rule was used to quantitatively simulate the interfacial interaction energy between different rough flocs and the polyvinylidene fluoride (PVDF) membrane. The results obtained from the combination strategy and the traditional XDLVO method were compared, and the measured interaction energy of smooth interface was compared with that obtained from theoretical simulation. The comparison showed that the combination strategy is suitable to simulate the interfacial interaction energy of flocs in a system of coagulation–membrane filtration. Meanwhile, the roughness of flocs will lead to a difference of 1–2 orders of simulated magnitude in the interfacial interaction energy. Besides, the rough flocs fit the membrane fouling trend better than the smooth flocs. In other words, rough flocs’ surface morphology can introduce interactions between flocs and membrane interface, facilitating a higher degree of confidence in characterizing the tendency of membrane fouling [68].

In membrane bioreactors, the interfacial interactions determine the adhesion and membrane fouling caused by pollutants. Therefore, it is of great significance to propose an effective method to quantitatively analyze interfacial interactions [69]. The RBF and ANN were used to predict the interfacial interactions between rough film surfaces. The interaction data were quantified by the XDLVO method and used as the training samples for the RBF network. The results showed that, under the same conditions, the RBF neural network only needed about 1/50 of the calculation time compared to the advanced XDLVO method to obtain the predictions, indicating the higher prediction efficiency of the RBF neural network [70]. Meanwhile, the RBF neural network produced reliable results with acceptable accuracy, suggesting its broad application prospects in the study of membrane fouling and interfacial behaviors.

Specific flux is the main index of membrane permeability. It is defined as:

(7) $$J=J_{20}\cdot 1.025^{\left(T-20\right)}$$

(8) $$\mathrm{SF}=J_{20}/\Delta P$$

where, $J_{20}$ is the membrane flux at 20 °C, L/(m^2^ h); *T* is the temperature in °C; and SF is the specific flux or permeability of the membrane, L/(m^2^.h. kPa).

Xu et al. [71] used the specific flux decay rate with cumulative water yield to evaluate membrane life and established a strategy for membrane life prediction from two perspectives, namely the decline of average specific flux in an actual operation and the recovery of membrane permeability after off-line cleaning. In an actual operation of a MBR, when the average specific flux remains too low to meet the requirement of water production for a long time, the membrane is considered to have reached its service life, and membrane replacements will be necessary. The membrane life predicted by this method is denoted as T_life_-1. In an MBR operation, membrane fouling exacerbates gradually, and off-line cleaning is the main means to remove it and restore membrane permeability. If the specific fluxes before and after off-line cleaning remain the same, then it can be concluded that cleaning failed to restore membrane permeability; in other words, the membrane life has come to an end. The membrane life prediction is denoted as T_life_-2. By analyzing the long-term operation (>3 years) of three large-scale municipal wastewater MBR treatment projects (>10,000 m^3^/d) and the effects of NaOCl off-line cleaning, two methods were proposed to predict the membrane life: one was based on the descending trend of actual average specific flux and the other was based on the recovery of membrane permeability before and after off-line cleaning. The results showed that for a specific MBR project, T_life_-2 was slightly larger than T_life_-1, but when the membrane operation time became longer than T_life_-1, the actual clean water yield could not meet the requirements. Therefore, T_life_-1 has more practical engineering significance.

Fenu et al. [22] took a large-scale MBR project as the research object and tried to evaluate the membrane life from different perspectives, such as the failure of water production to meet the designated requirements, the failure of membrane permeability restoration, the maximum strength of membrane contact with the cleaning agent, the continuous increase of operation costs, and the continuous decline of mechanical strength. However, the feasibility and practical application value of Fenu’s model need further study.

**Figure 3:**
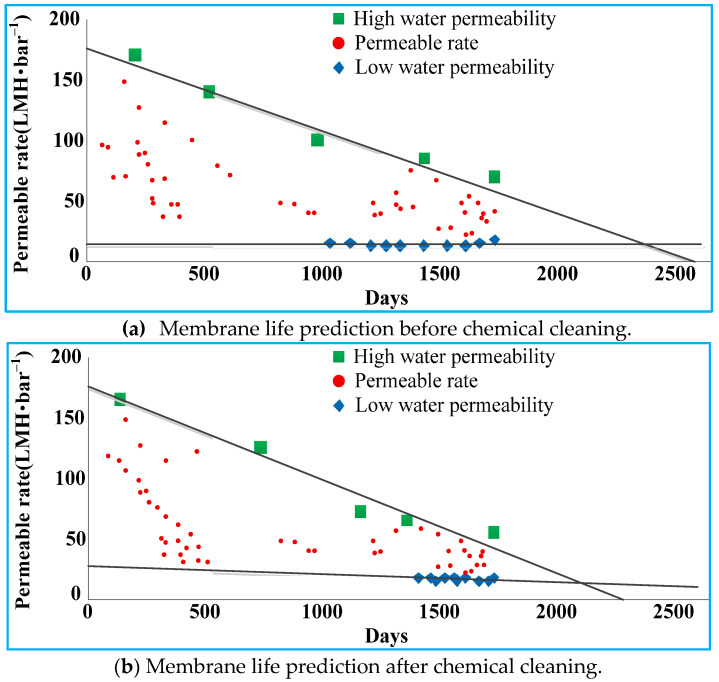
Membrane life prediction.

Wang et al. [72] drew a prediction chart for membrane life in a reclaimed MBR water plant (Figure 3). As the efficiency of chemical cleaning becomes lower and lower, the distance between the upper and the lower curves gradually shrinks and eventually intersects at a point at which a larger flux cannot be obtained by increasing the operating pressure, nor can the water permeability be further recovered by chemical cleaning. Therefore, this intersection point can be regarded as the end of the membrane life in a practical sense. The membrane life was first defined via different concepts, and it was predicted from various perspectives such as water yield, water quality, decay of water permeability, cumulative chlorine contact value, and membrane performance. The results showed that permeability decay and membrane performance are feasible parameters to judge membrane life, while the remaining three parameters are not ideal.

Various analytical tools were used to systematically study the changes in the removal of chemical oxygen demand (COD), biogas generation, sludge and cake properties, and their correlation with membrane fouling before and after changes to the pH of the feed solution [73]. The results showed that when pH was 8.0, the COD removal rate, gas production rate, and membrane filtration performance of submerged anaerobic membrane bioreactor (SAnMBR) did not show significant alteration. However, when pH was 9.1 or 10.0, the three variables showed a significant change, which lasted for a long time. An increase of pH will lead to the dispersion of sludge flocs and the accumulation of colloids, solutes, and biopolymers in the sludge suspension, resulting in lower membrane performance. Statistical analysis showed that the ratio of protein (PN) to polysaccharide (PS) in extra-cellular polymeric substance (EPS) had a strongly negative correlation with the membrane fouling rate. When pH was 10, under such an alkaline environment, the particles deposited on the membrane surface became smaller and condensed into a more compact cake-like layer, resulting in a higher rate of membrane fouling.

An improved collision adhesion model has been adopted to study the interactions between algae interfaces and hydrodynamic forces without neglecting any hydrodynamic force on the algae. The TOPSIS (technique for order preference by similarity to an ideal solution)—GRA (grey relational analysis) method was also used to evaluate membrane fouling performance, which was studied and predicted by combining the improved collision adhesion model with the back propagation neural network (GA-BP) optimized by a genetic algorithm [74]. The concept of “critical vibration frequency” was put forward, and the energy consumption to separate algae was analyzed, providing theoretical guidance for real-world practices.

Park et al. [24] studied a vertically-oriented hollow fiber membrane module in a pilot-scale bioreactor. The module was equipped with two aerators for simultaneous air injections, one facing downward and another facing upward. Different air jet structures would have significant impacts on the features of membrane fouling, and the experimental results showed that air injection both upward and downward could alleviate membrane fouling effectively. Besides, membrane permeability was revealed to be related to the dynamic information of the variables during membrane treatment. Therefore, further studies are worthwhile to find out whether such correlation can be used to predict membrane fouling.

In a pilot-scale membrane bioreactor (SMBR) for municipal wastewater treatment, the normalized permeability is negatively correlated to the MLSS concentration while positively correlated to the aeration intensity of coarse bubbles. As the concentration-time continued to extend, a small increase in MLSS concentration and mixture viscosity became more common at a certain MLSS concentration, resulting in lower membrane permeability [25].

Some scholars have successfully predicted the variation trend of membrane flux by studying the correlation between membrane flux and several variables in membrane filtration [26,27]. However, membrane fouling is a complex and dynamic process, and the numerous factors affecting membrane permeability and their mutual interactions make it difficult to describe membrane permeability with a simple variable relationship [75]. Martín-Pascual et al. [23] proposed a general mathematical model to estimate membrane permeability and corrected the model parameters with the actual measurements of the process variables. This model is advantageous due to fewer parameters involved and a simple correction process. It has been widely used in the calculation of membrane permeability. However, the parameters included in this model are rather inaccessible for online corrections, and the prediction accuracy is low.

Griffiths et al. [45] developed a mathematical model based on the adhesion probability of particles on the membrane pore wall and the probability of particles falling in a specific pore. In the model, a flat membrane was studied under constant pressure for total flow rate and permeation volume per unit membrane area. The relationship between the total flow rate and the permeation volume per unit membrane area was found to illustrate the main fouling mode at a certain time. Specifically, the fouling modes are standard plugging, in which a certain number of particles adhere to the pore wall and block the pores, and complete plugging, in which larger particles fall on the membrane surface, block the pores completely, and turn into a cake layer. Although this mathematical model can describe these different fouling modes, it cannot predict the TMP or the permeate flux at a given time.

Pimentel et al. [46] proposed a model to reproduce the dynamics of TMP in submerged MBR with infiltration flow rate, aeration rate, solid concentration, and temperature as the inputs. This dynamic model could predict the evolution of TMP for an acceptable period (about 10 days), and the determination coefficient between the predicted TMP and the experimental data was about 0.95. However, the model could not predict TMP evolution in real-time. Charfi et al. [48] studied the anaerobic fluidized bed MBR with granular activated carbon (GAC) and proposed several semi-empirical mathematical models to predict the parameter dynamics, including the dynamics of MLSS concentration or flux. These models could describe the variations of TMP effectively, and its R^2^ varied from 95.63% to 99.93%. Besides, they could predict membrane fouling in an anaerobic fluidized bed MBR. Judging from these studies, it could be concluded that as long as the number of input parameters is limited, these mathematical models can effectively predict membrane fouling.

In the field of membrane technologies, it is very difficult to study the internal fluid flow of a membrane module, and one feasible strategy to tackle the problem is to apply computational fluid dynamics (CFD). TMP evolution was simulated in a pilot-scale anaerobic MBR equipped with a flat membrane by ANSYS software. However, membrane optimization by CFD increases the computational cost. Nevertheless, the integration of artificial intelligence (AI) and CFD could facilitate simulation of the membranes and the phase separation process. Babanezhad et al. [76] used the adaptive-network-based fuzzy inference system (ANFIS) model with different parameters to learn about membrane technology. The purpose of the study was to find out how to adjust different parameters in the ANFIS model to improve the prediction accuracy of the AI model on membrane performance. The results indicated that the AI algorithm will obtain a high prediction performance with more input variables. That is, to predict the RUL, the more input variables, the higher the prediction accuracy. When the number of inputs increased to 5, the R-value of the AI predictions increased to 0.99, indicating high accuracy of the AI algorithm in predicting membrane performances.

Recently, Zhang et al. [49] developed a 3D CFD model for open field operation and manipulation (OpenFOAM^®^) to predict the evolution of permeation flux in MF tubular membranes. The membranes were equipped with baffles as turbulence promoters to reduce particle depositions on the membrane surface. The model produced satisfactory simulations of the MF process. However, CFD has some limitations. The numerical calculation algorithms used in CFD simulation software are based on several selected mathematical models. If a large number of inputs are considered, these models will be too complex to be developed. Besides, the research on CFD prediction of membrane fouling has predominantly focused on membranes with a simple geometric structure. Relevant articles have put forward several basic assumptions to simplify the numerical resolution, which weakens their practical value.

The mathematical models, including the CFD models, are of great significance for the prediction of membrane fouling. However, membrane fouling is a complex phenomenon that is dependent on many factors such as the concentration of extra-cellular polymeric substances and soluble microbial products, which are related to the operation parameters, including biological activity and aeration. Therefore, if fewer parameters are considered and some approximations and assumptions are made to the treatment system, these mathematical models could be calculated with simple equations to simulate the fouling mechanism without complex implementations.

## 4. Conclusions

MBR plays an important role in wastewater treatment because of its excellent performance. However, the problem of membrane fouling restricts the application of MBR to a great extent. As the technologies for wastewater treatment advance, the requirements for wastewater treatment become higher, and the control of membrane fouling is becoming recognized as the key to overcome the bottleneck of MBR developments. Real-time, fast, and accurate predictions of membrane fouling can not only enhance its control but also reduce the operating costs and improve the efficiency of wastewater treatment. To predict the evolution of membrane resistance, many mathematical methods based on membrane fouling mechanisms have been developed. However, due to the complexity of membrane fouling, these methods remain quite limited, and many assumptions are necessary to simplify them in order to make them feasible for calculations. Artificial neural network was first applied to the predictions of factors related to membrane fouling due to its good modeling capability, and indeed achieved satisfactory results in a very short time. It has also been used to establish an input-output prediction model based on practical membrane fouling data. However, since membrane fouling mechanisms are complex and it is difficult to collect data, the establishment of an ANN-based membrane fouling prediction model still faces many challenges. To solve the problems mentioned above, further research should focus on the following aspects:

(1) Membrane fouling mechanisms in MBRs of different structures and scales should be studied. An important premise of accurate and rapid membrane fouling prediction is the thorough understanding of the underlying mechanisms. Meanwhile, the development of an accurate and real-time online collection system of membrane fouling data can help to build a more comprehensive prediction model with higher prediction accuracy.
(2) Further research should focus on remaining useful life (RUL) prediction of the membrane modules at various failure modes. Most of the current research has focused on the residual life prediction at a single failure mode, ignoring that the failure of the membrane modules is usually caused by the synergistic effect of multiple failure modes. Under certain external impacts, the membranes could suddenly fail to provide normal functions. Therefore, residual life prediction at various failure modes is worthy of further study.
(3) Intelligent feature extraction and remaining useful life prediction should be addressed in future research. An accurate prediction of remaining useful life of membrane components is dependent on the extraction of effective information from the large amount of data obtained from monitoring. However, traditional extraction methods for statistical data and shallow machine learning strategies need to rely on a large number of signal data and expert experience to extract the feature information manually. When processing a large amount of monitoring data from complex engineering equipment, these subjective data extraction methods are seriously limited. Deep learning, such as deep belief network and convolutional neural network, can overcome such problems to some extent, but relevant research is still scarce, suggesting the necessity for further research.
